# MAPPING ASSISTIVE TECHNOLOGIES ON THE PROGRESSION OF ALZHEIMER’S DISEASE: A SCOPING REVIEW

**DOI:** 10.1093/geroni/igae098.2590

**Published:** 2024-12-31

**Authors:** Nadia Mirjan, Aleksandra Zecevic

**Affiliations:** University of Western Ontario, London, Ontario, Canada; University of Western Ontario, London, Ontario, Canada

## Abstract

A growing population of people living with Alzheimer’s Disease urges improved support for aging in place and with dignity. Assistive technologies (ATs) can be used to delay institutionalization, reduce caregiver burden, and improve quality of life for this population and their care partners. The abilities and needs of this population change during disease progression, requiring a better understanding of which ATs could be used at each stage of the disease. The purpose of this scoping review is to generate knowledge on how ATs can be mapped on seven stages of Alzheimer’s Disease progression described by the Global Deterioration Scale. The review follows the Arksey and O’Malley framework to identify and harvest information from Medline OVID, Scopus, CINAHL, and Embase OVID databases. Inclusion criteria were Alzheimer’s Disease, technology interventions of any type and duration, English language, and the time frame between 2000-2023. Data was extracted and thematically analyzed using six predetermined domains of ATs for dementia, namely safety devices, clinical devices, memory aids, ATs for preventing social isolation, ATs for leisure activities, and ATs for supporting everyday tasks. A total of 53 articles were included. Findings show that a variety of ATs are available throughout the disease progression. High technology (e.g., tracking devices) mainly target early stages, while low technology (e.g., weighted blanket) target later stages. Some, such as music therapy, are present at every stage of disease. The map has the potential to inform people with dementia, care partners, technology companies, distributors, policy makers and service providers.

